# Hospital clowning: a paediatrician’s view

**DOI:** 10.1007/s00431-016-2821-8

**Published:** 2016-12-24

**Authors:** Lennard T. van Venrooij, Pieter C. Barnhoorn

**Affiliations:** Department of Public Health and Primary Care, Leiden University Medical Center (LUMC), Albinusdreef 2, 2333 ZA Leiden, the Netherlands

**Keywords:** Paediatricians, Paediatric residents, Hospital clowns, Perceptions, Focus group

## Abstract

This study investigates the current position of hospital clowns from the perspective of paediatricians and paediatric residents. A total of 14 attending paediatricians and paediatric residents participated in two focus group sessions. Data were analysed using Atlas.ti 5.0. In general, physicians reported positive experiences regarding the interaction between hospital clowns and paediatric patients on the ward. Physicians were more interested in research on children’s perception of hospital clowns than in research on the clinical efficacy of hospital clowning. No direct collaboration between physicians and hospital clowns was reported. However, physicians proposed conditions which may streamline their encounters with hospital clowns such as clear communication prior to hospital clown visits, and the condition that visits do not impede medical interventions.

*Conclusion*: Overall, paediatricians and paediatric residents view the positive impact on paediatric patients as the most important aspect of hospital clown visits, rather than the clinical efficacy of hospital clowning. In light of the growing number of hospital clowns worldwide, this article provides recommendations for arranging their encounters with paediatricians and paediatric residents to maintain optimal health care.

**Table:** 

**What is known:** • *Previous studies show a clinically significant pain- and anxiety-reducing effect of hospital clowning in paediatric patients admitted to hospitals or undergoing (invasive) medical procedures.* • *In general, paediatricians have positive ideas about hospital clowns, aside from personal prejudices.*
**What is new:** • *This novel study gives deeper insight into day-to-day interaction between paediatricians and hospital clowns on the ward*. • *This study provides recommendations for clinical practice to arrange encounters between physicians and hospital clowns during hospital clown visits*.

## Introduction

The hospital clown has existed in many different cultures since the end of the twentieth century [9]. Nowadays, there is a rapidly growing number of hospital clowns who work in paediatric settings worldwide [9, 16]. Modern hospital clowns use humour to achieve a personal and trusting atmosphere between hospital workers and patients in the clinic [9]. Hospital clowns aim to reduce stress, fear, helplessness and sadness in the hospitalised paediatric patient [2]. The effect of hospital clowning is divided into four levels: the physiological level (release of endorphins which stimulate the immune system), the emotional level (initiating positive feelings), the cognitive level (distraction from own situation) and the social level (stimulating social interaction between the hospital clown and the child) [2]. Randomised controlled trials among children who underwent various invasive procedures showed pain relief and anxiety reduction in children and parents before or during the intervention by means of the presence of a hospital clown [8, 12, 13, 21, 24]. Previous studies show that hospital clowns are appreciated by patients, parents and hospital staff [2]. Most physicians believe that hospital clowns can have a positive impact on the paediatric patient and its health, despite the fact that some physicians personally do not like hospital clowns [2, 3, 11].

The importance of efficient collaboration between physicians and nurses has been stressed in the literature, for example, its positive impact on patient outcomes in adult acute care, adult intensive care and in neonatal settings [1, 5, 15, 17, 19, 20]. Compared to how physicians and nurses perceive their collaboration, little is known about encounters between physicians and non-clinical hospital personnel, in particular hospital clowns [4]. Furthermore, in contrast to research which primarily focuses on the general reputation of hospital clowns, little research focuses on the day-to-day interaction and collaboration between physicians and hospital clowns. Because the increasing number of hospital clowns may give rise to more encounters on the ward, it is important to explore physicians’ perceptions of hospital clowns. Therefore, this study is focussed on the following research question: ‘*what is paediatricians and paediatric residents’ perception of the current position of hospital clowns?*’

## Materials and methods

This study includes paediatricians and paediatric residents of the paediatric departments of Haga Hospital The Hague and Leiden University Medical Center, the Netherlands. Every week, two hospital clowns are scheduled to entertain hospitalised children on the ward. During their visits, hospital clowns ask the pedagogic workers of the department which children to visit and which not to visit. Reasons (not) to visit a specific child include the child’s mood (e.g. a visit would make the child more sad, which is expected to have negative consequences for its recovery) and relevant clinical information (e.g. infection risk). If direct contact is not possible, children watch the hospital clowns play from a distance. After 10 min, the hospital clowns move on to another child. All visiting hospital clowns have completed a hospital clowning course, for which they have been selected based on stage experience, empathic concern and knowledge of nursing ill and disabled children.

Focus groups were chosen as the method of data collection. Focus group methodology is a form of qualitative research that can be used to explore subjects that are not well understood or poorly described. One of the most important advantages of focus groups is that the retrieved information can be interpreted in the context of a group. The choice of focus group is observer dependent. Different group configurations may impart a range of ideas and insights into a research question [22].

Using an interview guide, two 60-min semi-structured focus groups were conducted in the paediatrics departments of Haga Hospital The Hague and Leiden University Medical Center (Appendix 1). Both focus groups were audio recorded and transcribed verbatim. To achieve anonymity of data, participants were given alpha-numerical codes (e.g. 1A = participant A of focus group 1). An administrative specialist (DT), who acted as an independent focus group observer, took simultaneous observational field notes recording verbal and non-verbal information, including facial expressions, gestures of participants and overall body language. These observations were intended to add depth to the information and to enhance the accuracy and analysis of the information collected through dialogue recordings. These field notes were subsequently added to the verbatim transcripts prior to the content analysis.

Paediatricians and paediatric residents of Haga Hospital The Hague (focus group 1), the Netherlands, and Leiden University Medical Center (LUMC, focus group 2), the Netherlands, were chosen as the sample frame for this study. No strict in- or exclusion criteria for participation were formulated. An e-mail was sent to participants via the secretariats of both departments prior to participation, providing a brief summary of the study including the purpose of the study, participant requirements and confidentiality. A lunch was offered as a reward for participation. Participation was voluntary. The researchers (LV and PB) did not have a hierarchical relationship with the participants.

The questioning framework covered several questions (Appendix 1), which were inspired by the findings of previous surveys on hospital staff perceptions of hospital clowns. The research supervisor (PB) validated the questioning framework and all results to avoid bias and misinterpretation of the data.

### Data analysis

The study results were analysed using Atlas.ti 5.0. The analysis incorporated two phases. The first phase was characterised by identifying covering subthemes inductively. More than one subtheme per quote was possible. The second phase was characterised by assigning these subthemes to one or more of the questions in the question framework (domains). To explore common cognitions of the participants, the number of times a specific subtheme was mentioned per domain was counted. Because the results showed no further inducement, other methods of analysis were not used. The analysis was discussed with and reviewed by the research supervisor (PB).

## Results

Two focus group discussions were held with one paediatrician and eight paediatric residents in focus group 1 and two paediatricians and three paediatric residents in focus group 2 (*n* = 14). All physicians were female (100%), with an average age of 31 (24–43). Table 1 shows the demographic breakdown of the participating paediatricians and paediatric residents.

**Table 1:** Characteristics of paediatricians and paediatric residents (*n* = 14)

Demographic characteristics	Number of participants
Sex	
Female	14 (100%)
Age	
Mean age (±SD)	32.1 (±5.9)
Function^a^	
Paediatric residents	11 (79%)
Paediatricians	3 (21%)

^a^The Haga Hospital focus group (focus group 1) consisted of one paediatrician and eight paediatric residents. The Leiden University Medical Center focus group (focus group 2) consisted of two paediatricians and three paediatric residents

### Domain 1: paediatricians’ perception of the work of hospital clowns in general

First, we explored general cognitions, feelings and particular experiences with hospital clowns in the department. Overall, physicians were quite familiar with the work of hospital clowns in their department. Some physicians believed that hospital clowns facilitate stress reduction and distraction from the illness of the paediatric patient by providing some form of organised children’s entertainment. After being asked what ideas paediatricians have of the work of hospital clowns, one physician expressed her doubts on whether the figure of the clown is still current. Furthermore, four participants considered themselves afraid of clowns, although they had not been diagnosed with ‘coulrophobia’. According to some physicians, personal encounters with hospital clowns are scarce and of relatively short duration. “*You sporadically speak to them, but see them often. It is like two separated worlds*” *[1H]*.

Most physicians had positive experiences with hospital clown interactions with paediatric patients. Despite their personal views, physicians believed that most patients and their parents perceive hospital clowns positively. However, physicians also report that the reaction of the paediatric patient depends on the age and personality of the child. “*I think my feelings on them are less positive than the effect they have on children*” *[2C].*

As for negative experiences, one physician reported that hospital clowns sometimes make too much noise in the hallways. Furthermore, some physicians indicated that they avoid the hospital clowns, because they make them feel uncomfortable. These physicians mentioned that hospital clowns tend to keep in character, even if children are not around: *“...sometimes I see a complete stranger entering the room with a red cap on his or her nose. If I could make someone’s acquaintance before visiting the child, it would be easier for me to play along” [1A]; “I want to see the person behind the clown” [2G]*. Most physicians indicated that the job responsibilities of paediatricians or paediatric residents and hospital clowns have to remain separated.

### Domain 2: paediatricians’ perceptions of studies on the clinical efficacy of hospital clowning

Next, the focus groups probed physicians’ perceptions of studies on the clinical efficacy of hospital clowning, e.g. pain reduction in patients after surgical treatment. Because none of the physicians were familiar with the existence of such research, they were not convinced of a clinically beneficial effect. Additionally, some physicians were not interested in such research. Providing information on the research methodology and the results of existing studies during the interview did not alter participants’ opinions. An opinion shared among physicians was that more knowledge is needed on the way paediatric patients perceive hospital clown visits: *“I am more interested in studies on paediatric patients’ experiences with hospital clowns, rather than research on their clinical efficacy” [1A]. “How did they measure the effects? There are so many interventions that are conducted in a hospital which could bias the results. I would like to know how the authors are able to say that a positive effect is due to hospital clowns specifically” [2E].*

### Domain 3: paediatricians’ perceptions of their departmental procedure regarding hospital clown visits

Most physicians did not consider being involved in the procedure of their department regarding hospital clown visits to be part of their job responsibilities. Therefore, none of the physicians mentioned positive or negative personal experiences regarding this procedure. *“I have too many people I have to confer with. Being involved in this procedure would give me too heavy a workload” [1F]. “I think that being involved in this procedure is not part of our job responsibilities” [2D].*

Almost all physicians believed that nurses and pedagogic workers are the appropriate persons to arrange this procedure. According to physicians, nurses and pedagogic workers see hospital clowns more often and know the social context of the paediatric patient better than physicians do. Therefore, they are in the best position to provide feedback regarding patient care to physicians. Physicians note the advantages of being assisted by pedagogic workers (e.g. they experience that pedagogic workers know the way physicians think) but are not yet convinced that hospital clowns can provide the same advantages.

### Domain 4: paediatricians’ perceptions of direct collaboration between hospital clowns and physicians

All physicians indicated that no direct or indirect collaboration existed between physicians and hospital clowns in their department. Therefore, hypothetical direct collaboration was discussed, such as the Dream Doctors Project in Israel. In this project, hospital clowns are trained to work as part of multidisciplinary care teams in various medical units, which carry out procedures aimed at improving patient wellbeing and advancing care [6]. Some physicians responded quite positively, others were less positive. *“Maybe it is due to my education level, which is quite recent, but I think my duty is to adapt to the child and to do things right technically. I do not necessarily have to communicate with the hospital clowns. Hospital clown are part of the child’s inner world” [2D].*

Physicians stated four conditions for direct collaboration between hospital clowns and physicians. First, collaboration must lead to the soothing and distraction of a child. Second, collaboration must not impede medical intervention (e.g. by being too noisy). Third, collaboration must not lead to a child associating hospital clowns with tedious medical interventions. Fourth, clear communication between physicians and hospital clowns about the expectations of both parties regarding hospital clowns’ visits is mandatory.

Some physicians responded that close cooperation between hospital clowns and physicians could have a positive impact on medical intervention outcomes, provided that aforementioned conditions are met. *“I once attended a conference in which the audience discussed laughing gas during sedation. One of the outcomes of that discussion was that when the child is not comforted, laughing gas loses its effect. If a hospital clown could provide this comfort, then close collaboration between hospital clowns and physicians could be clinically effective” [2C].*

One physician suggested the inclusion of hospital clowns in a treatment plan. In response to this suggestion, some physicians stated that they wanted to get to know a particular hospital clown personally before deciding whether to include this hospital clown or not.

## Discussion

### Principal findings

In light of the rising numbers of hospital clowns, leading to more frequent encounters between hospital clowns and physicians, we qualitatively investigated the paediatricians and paediatric residents’ perceptions of hospital clowns. The results of the study can be seen as recommendations for medical institutions and hospital clowning services on how to organise hospital clown visits. Furthermore, the results can be a starting point for future research on encounters between hospital clowns and physicians on the ward.

### Relationship to other studies and the literature

Our findings showed that physicians generally have positive opinions on the effect of hospital clown visits on paediatric patients, despite their prejudices or negative personal opinions. This suggests that physicians consider their own perceptions of hospital clowning to be less important than their patients’ opinion, which is consistent with other studies on the position of hospital clowns from the perspective of hospital staff [2, 3, 11]. According to physicians, neither interaction with visiting hospital clowns nor facilitating the visits of the clowns on the ward is part of their job responsibilities. We were unable to find other studies that investigated the role physicians have in facilitating hospital clown visits on paediatric wards.

This study gave more insight into how physicians view studies on the clinical efficacy of hospital clowning. In contrast to such research, the physicians showed particular interest in research on the opinions of paediatric patients on hospital clowns. To the best of our knowledge, this finding was not reported in other literature. However, the opinions of paediatric patients, and notably the anxiety-reducing effect of hospital clown visits, have been described extensively [8, 12, 13, 21, 23, 24]. Furthermore, the finding that physicians are less interested in the clinical efficacy of hospital clowning is surprising, since recently more and more studies are published in which a beneficial clinical effect of hospital clowning is described [7, 18]. This may indicate that there is a discrepancy between the way physicians value the outcome of the work of hospital clowns (e.g. distracting the paediatric patient from his or her invasive procedure) and the way the outcomes are described in the literature (e.g. pain reduction after a treatment due to anxiety reduction). Other literature focuses on the beneficial effect hospital clowns have on the patient-physician relationship, sometimes beyond the moment of interaction. Physicians may use the impact of hospital clown visits on the child to improve their own relationship with the child and its family (e.g. by discussing mementos and photos left by the hospital clowns) [10].

Important insight was gained in the area of direct or indirect collaboration between physicians and hospital clowns on the ward. Physicians who participated in our study reported no existing collaboration with hospital clowns. However, one of the conditions physicians listed which would assure an efficient collaboration, clear communication, is mentioned in previous research as an element which can negatively impact the child’s overall experience in the hospital [11].

### Limitations and strengths—implications for future research

This study has limitations. Firstly, the participants of the focus groups are only a sample of the paediatrician and paediatric resident population as a whole. More focus groups consisting of paediatricians and paediatric residents of various departments could provide data regarding how to streamline their encounters with hospital clowns, which can also be applied to other wards. Secondly, all participants were female. In comparison, 53% of Dutch paediatrician are female [14]. Since it would be interesting to examine the topic on both male and female, future research should also include male paediatricians. Thirdly, the study population consisted of only three paediatricians, because other paediatricians who were approached had supervision tasks during the interviews.

This study also has several strengths. Firstly, we included a higher number of participants compared to similar studies [11]. Secondly, this study used a study methodology which explored paediatricians and paediatric residents’ perceptions in more detail than previously conducted studies, which were mainly surveys [2, 3].

### Implications for practice

Our findings give rise to the following implications for clinical practice:

- Sufficient clinical information must be given to hospital clowns prior to their visit so it can be decided which child will be visited and which will not. It is recommended that hospital clowns meet the present physician in person, without staying in character.
- Overall, physicians perceive hospital clowning to be beneficial to the paediatric patient. However, since they view their work as the primary treatment policy, hospital clowns are advised to adjust their act to the activities of the physician.
- Physicians should be encouraged to engage in dialogue with hospital clowns in order to further appreciate their role in the hospital stay of the paediatric patient.

## Conclusion

In general, physicians have positive experiences regarding how hospital clowns interact with paediatric patients and how children experience their act. However, physicians do not believe that facilitating the visits of clowns on the ward is part of their job responsibilities. Physicians are not aware of the existence of research on the clinical efficacy of hospital clowning and believe that research on how children experience hospital clowns is more important. There is no direct collaboration between physicians and hospital clowns on the ward, but physicians report some conditions which may streamline their encounters, such as clear communication prior to hospital clown visits and the condition that the visits must not lead to the impediment of medical interventions. Because we expect that physicians and hospital clowns will meet more and more in the future, additional research on how to arrange these encounters is needed.

## Appendix 1: Interview guide

### Informed consent

I hereby confirm that I have been adequately informed by the researcher about the nature and conduct of the study. I am aware that the results of the study will be anonymously processed into a research report. I understand that my participation is voluntary and that I may, at any stage, without prejudice, withdraw my consent and participation in the study.

### Demographic characteristics

- Q1. What is your gender?
- Q2. What is your age?
- Q3. What is your profession (paediatrician or paediatric resident)?

### Domain 1: Physicians’ general ideas on the work of hospital clowns

- Q4. Are you aware of the work of hospital clowns in general?
- Q5a. If so, what are your personal experiences with the work of hospital clowns on the ward?
- Q5b. If not, what is your idea of the work of hospital clowns?
- Q6a. What do you like about the work of hospital clowns?
- Q6b. What do you dislike about the work of hospital clowns?
- Q7. According to you, what aspect of the work of hospital clowns could be improved (in general)?

### Domain 2: Physicians’ knowledge of the clinical efficacy of hospital clowning

- Q8. Do you know anything about the scientific basis of hospital clowning in particular?
- Q8a. If so, what do you know about the scientific basis of hospital clowning in particular?
- Q8b. If not, skip to Q10.
- Q9. According to you, how convincing is the scientific basis for the clinical efficacy of hospital clowning?
- Q10. What would convince you of a beneficial clinical efficacy of hospital clowning?

### Domain 3: Paediatricians’ views on the departmental procedure regarding hospital clown visits

- Q11. What do you know about your department’s procedure regarding hospital clown visits?
- Q11a. What are your positive personal experiences regarding this procedure?
- Q11b. What are your negative personal experiences regarding this procedure?
- Q12. Are you involved in this procedure?
- Q12a. If not, do you want to be involved in this procedure?
- Q13. Which members of your department are involved in this procedure?
- Q14. According to you, which members of your department are the right persons to be involved in this procedure?
- Q15. Do you think that any aspect of your department’s procedure regarding hospital clown visits could be improved?

### Domain 4: Physicians’ thoughts on close cooperation between physicians and hospital clowns

- Q16. Do you think that there should be close cooperation between physicians and hospital clowns (e.g. the Dream Doctors Project in Israel)?
- Q16a. If so, why do you think so?
- Q16b. If not, why not?
- Q17. What could hospital clowns do to establish this cooperation?
- Q18. What could your department do to establish this cooperation?

***End of focus group interview.***

## Abbreviations

DT: Dieke van Tol (minutes secretary)
LUMC: Leiden University Medical Center, the Netherlands
LV: Lennard T. van Venrooij (primary author)
PB: Pieter C. Barnhoorn (co-author)
WMO: Medical Research Involving Human Subjects Act
[2H] (example): Anonymous reference to a specific participant, e.g. participant “H” from focus group “2”
